# Burden and trend of cardiovascular diseases among people under 20 years in China, Western Pacific region, and the world: An analysis of the global burden of disease study in 2019

**DOI:** 10.3389/fcvm.2023.1067072

**Published:** 2023-02-15

**Authors:** Yue Zhang, Changjian Lin, Ming Liu, Wei Zhang, Xiaoyun Xun, Jinyi Wu, Xiaopan Li, Zheng Luo

**Affiliations:** ^1^School of Public Health, Department of Epidemiology, Shanxi Medical University, Taiyuan, China; ^2^Department of Cardiovascular Medicine, Ruijin Hospital, Shanghai Jiao Tong University School of Medicine, Shanghai, China; ^3^Department of Health Management Center, Zhongshan Hospital, Shanghai Medical College of Fudan University, Shanghai, China; ^4^Shanghai Engineering Research Center of AI Technology for Cardiopulmonary Diseases, Zhongshan Hospital, Fudan University, Shanghai, China; ^5^Shanghai University of Medicine and Health Sciences Affiliated Zhoupu Hospital, Shanghai, China; ^6^Department of Public Health, Wuhan Fourth Hospital, Wuhan, China

**Keywords:** cardiovascular diseases, burden of disease, disability-adjusted life years, trend analysis, average annual percent change

## Abstract

**Objectives:**

Cardiovascular disease (CVD) is a global public health concern, but its disease burden and trend have been poorly studied in people younger than 20 years. This study aimed to fill this gap by evaluating the CVD burden and trend in China, Western Pacific Region, and the world from 1990 to 2019.

**Methods:**

We applied the 2019 Global Burden of Diseases (GBD) analytical tools to compare the incidence, mortality, and prevalence of CVD, years lived with disability (YLDs), years of life lost (YLLs), and disability-adjusted life years (DALYs) among people younger than 20 years from 1990 to 2019 in China, the Western Pacific Region, and the world. The trends of disease burden between 1990 and 2019 evaluated using the average annual percent change (AAPC) and the 95% uncertainty interval (UI) were reported.

**Results:**

Globally, in 2019, there were 2.37 (95% UI: 1.82 to 3.05) million incidence of CVD, 16.85 (95% UI: 12.56 to 22.03) million prevalence of CVD, and 74386.73 (95% UI: 64543.82 to 86310.24) deaths due to CVD among people under 20 years of age. The trends for DALYs decreased among children and adolescents in China, Western Pacific Region, and the world (AAPC = −4.29, 95% CI: −4.38% to −4.20%; AAPC = −3.37, 95% CI: −3.48% to −3.26%; AAPC = −2.17, 95% CI: −2.24% to −2.09%; *p* < 0.001, respectively) between 1990 and 2019. With the increase in age, the AAPC values of mortality, YLLs, and DALYs showed a notable downward trend. The AAPC values of mortality, YLLs, and DALYs in female patients were significantly greater than those in male patients. For all subtypes of CVD, the AAPC values showed a downward trend, with the largest reduction observed for stroke. From 1990 to 2019, a decline in the DALY rate for all CVD risk factors was observed, with a significant decrease in environmental/occupational risk factors.

**Conclusion:**

Our study shows a decline in the burden and trend of CVD among people younger than 20 years, which reflects the success in reducing disability, premature death, and the early incidence of CVD. More effective and targeted preventive policies and interventions aimed at mitigating preventable CVD burden and addressing risk factors from childhood are urgently needed.

## Introduction

Cardiovascular diseases (CVDs) are chronic non-communicable diseases (NCDs) that seriously threaten human health. The global burden of disease (GBD) in 2019 shows that CVD is responsible for 18.5 million deaths worldwide, accounting for approximately 31% of total deaths (1). According to the statistics of China Cardiovascular Health and Disease Report 2020 (2), the number of patients with CVD in China is up to 330 million, and the number of deaths is 4.58 million, which accounts for more than 40%, ranking the first in the total cause of death, and two out of every five deaths are caused by CVD. The Sustainable Development Goals (SDGs) released the target of a reduction in premature mortality owing to NCDs by one-third by 2030, and the State Council of China subsequently endorsed an important document aimed at reducing the age-standardized mortality rate of CVD in 2015 by 15% by 2025 (3). Thus, a consistent, comparable, and comprehensive analysis of the long-term trend at the global, regional, and national levels is essential to guide public policy and provide resource allocation for decision-makers.

Childhood and adolescence are vulnerable periods and a key window for adult health. However, a large number of studies have found that the incidence of CVD is scarce over this period (4). The majority of CVDs manifest in the middle age and beyond, and it is now clear that their origin may be during childhood and adolescence. Many traditional risk factors for CVD, such as hypertension, dyslipidemia, obesity, unhealthy diet, and smoking, begin during childhood and then tend to accumulate and increase with age. Social risk factors, such as low socioeconomic status and poor education, also increase the risk of developing CVD at a very young age. Research suggests very few adolescents, including children, have an ideal CVD health profile (5). Therefore, it is of great public health importance to focus on the burden and trend of CVD during this period. In addition, children and adolescents with an underlying heart disease, either congenital or acquired, deserve special consideration with regard to future CVDs. A report showed that congenital heart disease accounts for nearly one-third of all congenital birth defects, and a focus on congenital heart disease is indispensable to eliminating preventable child deaths and additional injury to the coronary arteries in the era of the SDGs (6). Given the enormous disease burden and health resource constraints, to stem the tide of CVD requires a life course approach with a preventive strategy starting from early childhood and adolescence.

The Global Burden of Diseases, Injuries, and Risk Factors Study 2019 (GBD 2019) framework, with a broad collection of data sources and statistical modeling approaches, enables the comparable assessment of CVD burden in terms of incidence, mortality, years of life lost (YLLs), years lived with disability (YLDs), and disability-adjusted life years (DALYs). GBD also provides information to understand both the trends in risk exposure and the trends in burden attributable to risks. Currently, the burden of CVD is studied predominantly in adults, with few studies in children and adolescents, and even fewer studies on the attributable disease burden. In this study, we aimed to estimate the burden of CVD and its trend among people under the age of 20 years, stratified by gender and subtypes, as well as CVD-related DALYs associated with potentially modifiable behavioral, environmental, and metabolic risk factors from 1990 to 2019 in the world, the Western Pacific Region, and China by using the data from the GBD 2019 study.

## Methods

### Data sources and definitions

Data for this study were obtained from the GBD 2019 study. The GBD 2019 generates estimates due to 369 diseases and injuries, 87 risk factors, and a combination of risk factors in 204 countries and territories. The details of the GBD 2019 eligibility criteria, the literature search strategy, and data extraction have been published elsewhere (7–9). We produced standard epidemiological measures such as incidence, prevalence, and mortality, as well as summary measures of health (YLLs, YLDs, and DALYs) among people younger than 20 years due to CVD. YLL is a measure of premature death within a group of people. YLD measures the amount of time people lose due to diseases and injuries that reduce their health but do not cause death. DALY is a comprehensive indicator to assess the disease burden of disability and premature death, which is obtained by adding YLLs and YLDs. In our analyses, CVD subtypes included hypertensive heart disease, ischemic heart disease, non-rheumatic valvular heart disease, rheumatic heart disease, stroke, aortic aneurysm, cardiomyopathy and myocarditis, endocarditis, and other cardiovascular and circulatory diseases. Three major risk factors, such as behavioral, environmental/occupational, and metabolic risk factors, were considered to be associated with CVD in GBD 2019. We covered four age groups: less than 5 years, 5–9 years, 10–14 years, and 15–19 years. To ensure transparency and replicability, our study follows the Guidelines for Accurate and Transparent Health Estimates Reporting (GATHER) (10). All data used in this study were obtained from the Institute for Health Metrics and Evaluation (IHME) website.

### Statistical analyses

We computed counts, rates (per 100,000), and DALYs to quantify the burden of CVD stratified by sex, age, and subtypes of people under 20 years. The trends of disease burden between 1990 and 2019 were evaluated using average annual percent change (AAPC), which was calculated by the Joinpoint Regression Program (Version 4.9.0.0. March 2021) (11). For each estimated metric, the 95% uncertainty interval (UI) was reported. 95% UI was calculated by taking 1,000 draws from the posterior distribution of each quantity, using the 2.5th and 97.5th ordered draw of the uncertainty distribution (12). We considered a *p*-value of <0.05 to be significant.

## Results

**Figure 1:**
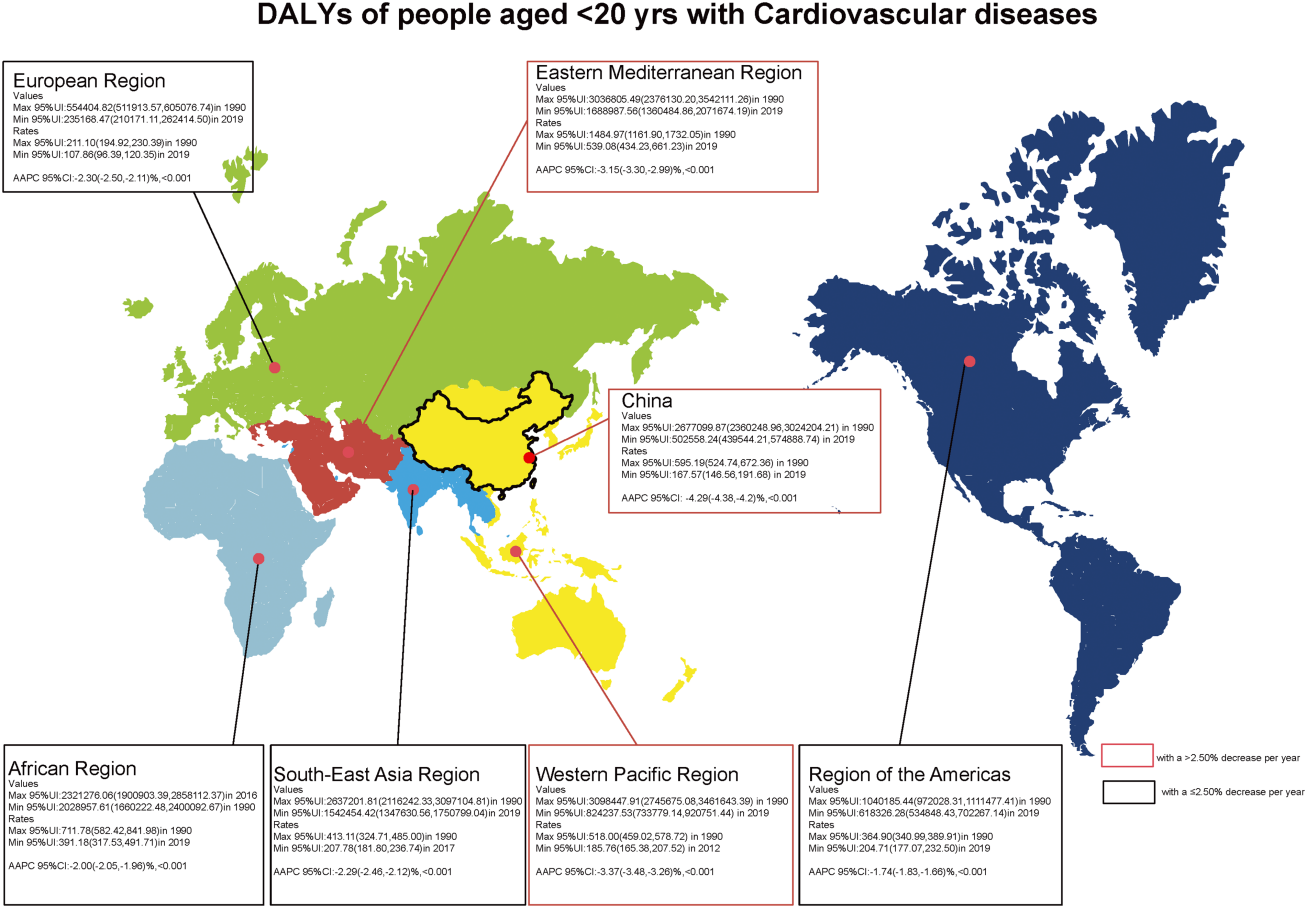
Number, rate, and average annual percentage change (AAPC) of DALYs among people with CVD 20  years.

The absolute number and rate of incidence, prevalence, deaths, YLLs, YLDs, DALYs, and AAPC from 1990 to 2019 caused by CVD in people younger than 20 years in China, the Western Pacific Region, and the world are shown in Table 1. From 1990 to 2019, the absolute number, rate of incidence, and prevalence of CVD and also deaths due to CVD among people under 20 years of age in China, the Western Pacific Region, and the world showed a continuous declining trend. Globally in 2019, there were 2.37 million (95% UI 1.82 to 3.05) incidence of CVD, 16.85 million (95% UI 12.56 to 22.03) prevalence of CVD, and 74386.73 (95% UI 64543.82 to 86310.24) deaths due to CVD among people under 20 years of age. The incidence of cases of CVD has almost become half from 409300.76 (95% UI 327352.27 to 504066.70) in 1990 to 203195.69 (95% UI 160256.44 to 255326.61) in 2019 in China. In 2019, rates on YLLs, YLDs, and DALYs for CVD were 228.33/100,000, 49.91/100,000, and 278.24/100,000, respectively, globally. The trends of DALYs and YLLs also decreased in children and adolescents in China, Western Pacific Region, and the world. At the global level, the absolute number of DALYs due to CVD in 1990 (12.39 million [95% UI 10.78–13.77]) significantly exceeded that in 2019 (7.17 million [6.26–8.25]). The disease burden of CVD among people under 20 years of age in China is lower than that in the global and Western Pacific Regions. From 1990 to 2019, the AAPC of mortality due to CVD among people younger than 20 years in China, the Western Pacific Region, and the world decreased by 4.79% (95% CI: −4.89% to −4.69%, *p* < 0.001), 3.73% (95%CI: −3.83% to −3.62%, *p* < 0.001), and 2.46% (95% CI: −2.53% to −2.38%, *p* < 0.001), respectively. The YLLs all showed a downward trend (*p* < 0.001), while DALYs due to CVD decreased in China, the Western Pacific Region, and the world (AAPC = −4.29, 95% CI: −4.38% to −4.20%; AAPC = −3.37, 95% CI: −3.48% to −3.26%; AAPC = −2.17, 95% CI: −2.24% to −2.09%; *p* < 0.001, respectively) between 1990 and 2019. Figure 1 intuitively shows the DALY values of CVD among people under the age of 20 years in different WHO regions. It was found that the AAPC values of CVD in the Western Pacific Region, the Eastern Mediterranean Region, and China have decreased by more than 2.5%.

**Table 1:** Absolute number and rate of incidence, prevalence, deaths, YLLs, YLDs, and DALYs due to CVD among people younger than 20 years and APCC for 1990–2019 in China, the Western Pacific Region, and the world.

Metrics		China		Western Pacific Region		Global	
		1990	2019	1990	2019	1990	2019
Incidence (95%UI)	Absolute number	409300.76	203195.69	475659.93	281124.2	1827746.25	2373195.97
		(327352.27,504066.70)	(160256.44,255326.61)	(381650.78,581603.92)	(223730.33,350821.12)	(1450692.42,2294789.46)	(1829940.75,3051990.37)
	Rate, per 100,000 people	91	67.75	79.52	63.36	80.39	92.01
		(72.78,112.07)	(53.43,85.13)	(63.80,97.23)	(50.42,79.07)	(63.81,100.93)	(70.95,118.33)
	% (AAPC, 95%CI)	−0.87(−1.03, −0.71)^*^		−0.65(−0.80, −0.51)^*^		0.63 (0.55, 0.71)^*^	
Prevalence (95%UI)	Absolute number	2178911.814	1263148.913	2551936.98	1740261.8	11727242.46	16858400.17
		(1617433.26,2905474.49)	(932371.44,1675385.97)	(1906707.59,3355515.46)	(1304688.62,2272508.71)	(8854546.46,15263375.59)	(12565499.27,22032406.43)
	Rate, per 100,000 people	484.43	421.17	426.63	392.21	515.81	653.61
		(359.60,645.96)	(310.88,558.62)	(318.76,560.98)	(294.05,512.17)	(389.46,671.34)	(487.17,854.21)
	%, (AAPC, 95%CI)	0.21(−0.10,0.53)		0.30 (0.03,0.57)		1.07 (0.99,1.16)^*^	
Deaths (95%UI)	Absolute number	30135.18	4835.28	34787.46	8,250	139668.54	74386.73
		(26396.41,33960.60)	(4210.49,5513.12)	(30822.47,38943.28)	(7409.09,9156.37)	(121153.25,155316.95)	(64543.82,86310.24)
	Rate, per 100,000 people	6.7	1.61	5.82	1.86	6.14	2.88
		(5.87,7.55)	(1.40,1.84)	(5.15,6.51)	(1.67,2.06)	(5.33,6.83)	(2.50,3.35)
	%, (AAPC, 95%CI)	−4.79(−4.89, −4.69)^*^		−3.73(−3.83, −3.62)^*^		−2.46 (−2.53, −2.38)^*^	
YLLs (95%UI)	Absolute number	2472750.926	379256.3768	2847258.14	647555.03	11423222.51	5889324.54
		(2158901.23,2797897.97)	(330625.85,433093.92)	(2516179.36,3198558.80)	(581496.01,718859.32)	(9817748.43,12759023.73)	(5082198.99,6853521.31)
	Rate, per 100,000 people	549.75	126.45	476.01	145.94	502.44	228.33
		(479.98,622.04)	(110.24,144.41)	(420.66,534.74)	(131.06,162.01)	(431.82,561.19)	(197.04,265.72)
	% (AAPC, 95%CI)	−4.95(−5.06, −4.85)^*^		−3.89(−4.00, −3.78)^*^		−2.55(−2.63, −2.47)	
YLDs (95%UI)	Absolute number	204348.9393	123301.8668	251189.77	176682.5	968145.51	1287283.25
		(139843.14,287175.38)	(84632.77,173058.83)	(173158.12,349811.63)	(121704.92,244895.39)	(655710.36,1348218.25)	(856137.21,1805917.41)
	Rate, per 100,000 people	45.43	41.11	41.99	39.82	42.58	49.91
		(31.09,63.85)	(28.22,57.70)	(28.95,58.48)	(27.43,55.19)	(28.84,59.30)	(33.19,70.02)
	% (AAPC, 95%CI)	0.06(−0.13,0.25)		0.15(−0.01,0.31)		0.72 (0.66,0.78)	
DALYs (95%UI)	Absolute number	2677099.87	502558.24	3098447.91	824237.53	12391368.02	7176607.79
		(2360248.96,3024204.21)	(439544.21,574888.74)	(2745675.08,3461643.39)	(733779.14,920751.44)	(10781927.39,13776519.04)	(6263462.47,8259236.44)
	Rate, per 100,000 people	595.19	167.57	518	185.76	545.02	278.24
		(524.74,672.36)	(146.56,191.68)	(459.02,578.72)	(165.38,207.52)	(474.23,605.94)	(242.84,320.22)
	% (AAPC, 95%CI)	−4.29(−4.38, −4.20)^*^		−3.37(−3.48, −3.26)^*^		−2.17(−2.24, −2.09)^*^	

*p < 0.001, DALYs, disability-adjusted life years; YLDs, years lived with disability; YLLs, years of life lost; UI, uncertainty interval; CI, confidence interval.

Table 2 shows the AAPC of incidence, prevalence, deaths, YLLs, YLDs, and DALYs due to CVD among people younger than 20 years during 1990–2019 in China, the Western Pacific Region, and the world. With the increase in age, the values of AAPC of mortality, YLLs, and DALYs showed a notable downward trend. From 1990 to 2019, global DALYs from CVD decreased by 3.33%, 1.87%, 0.90%, and 1.09% annually among people under 5 years, 5–9 years, 10–14 years, and 15–19 years, respectively, but at the global level, the AAPC of the incidence and prevalence of CVD was observed to increase from 1990 to 2019. Compared with the global and Western Pacific Regions, the AAPC of DALYs in children under 20 years of age in China decreased the fastest (*p* < 0.001).

**Table 2:** Average annual percentage change (AAPC) of incidence, prevalence, deaths, YLLs, YLDs, and DALYs due to CVD among people younger than 20  years for 1990–2019 in China, Western Pacific Region, and the world.

	<5 years old		5–9 years old		10–14 years old		15–19 years old	
	AAPC 95%CI (%)	*P*	AAPC 95%CI (%)	*P*	AAPC 95%CI (%)	*P*	AAPC 95%CI (%)	*P*
**China**								
Incidence	−1.81(−1.95, −1.66)	<0.001	−0.82(−1.03, −0.62)	<0.001	−0.41(−0.68, −0.15)	0.003	−0.57(−0.75, −0.38)	<0.001
Mortality	−6.82(−7.11, −6.53)	<0.001	−4.42(−4.89, −3.94)	<0.001	−3.45(−3.93, −2.97)	<0.001	−2.85(−2.95, −2.74)	<0.001
Prevalence	−0.04(−0.37,0.29)	0.811	0.27(−0.00,0.54)	0.052	0.41 (0.10,0.72)	0.012	0.00(−0.38,0.39)	0.983
YLLs	−6.82(−7.11, −6.52)	<0.001	−4.41(−4.89, −3.94)	<0.001	−3.46(−3.94, −2.98)	<0.001	−2.85(−2.95, −2.75)	<0.001
YLDs	−0.16(−0.31, −0.02)	0.028	0.10(−0.09,0.28)	0.288	0.18(−0.04,0.39)	0.108	−0.09(−0.34,0.17)	0.486
DALYs	−6.69(−6.97, −6.42)	<0.001	−3.47(−3.81, −3.12)	<0.001	−2.22(−2.50, −1.94)	<0.001	−2.35(−2.48, −2.23)	<0.001
**Western Pacific Region**								
Incidence	−1.45(−1.58, −1.31)	<0.001	−0.62(−0.80, −0.44)	<0.001	−0.27(−0.47, −0.07)	0.009	−0.39(−0.55, −0.24)	<0.001
Mortality	−5.74(−5.90, −5.58)	<0.001	−3.32(−3.64, −3.00)	<0.001	−2.06(−2.38, −1.74)	<0.001	−1.96(−2.10, −1.82)	<0.001
Prevalence	0.28 (0.01,0.56)	0.040	0.31 (0.08,0.53)	0.009	0.39 (0.16, 0.63)	0.002	0.17(−0.14, 0.48)	0.279
YLLs	−5.75(−5.91, −5.58)	<0.001	−3.33(−3.65, −3.01)	<0.001	−2.06(−2.38, −1.74)	<0.001	−1.97(−2.11, −1.83)	<0.001
YLDs	0.02(−0.09, 0.12)	0.767	0.16 (0.02,0.31)	0.025	0.20 (0.05, 0.36)	0.014	0.05(−0.15, 0.26)	0.590
DALYs	−5.64(−5.78, −5.49)	<0.001	−2.61(−2.84, −2.38)	<0.001	−1.35(−1.55, −1.16)	<0.001	−1.61(−1.77, −1.45)	<0.001
**Global**								
Incidence	0.20 (0.15, 0.24)	<0.001	0.69 (0.57, 0.81)	<0.001	0.89 (0.73, 1.05)	<0.001	0.52 (0.45, 0.60)	<0.001
Mortality	−3.40(−3.49, −3.31)	<0.001	−2.49(−2.58, −2.39)	<0.001	−1.47(−1.58, −1.36)	<0.001	−1.44(−1.51, −1.37)	<0.001
Prevalence	0.98 (0.90, 1.05)	<0.001	0.97 (0.86, 1.08)	<0.001	0.98 (0.83, 1.12)	<0.001	0.92 (0.80, 1.05)	<0.001
YLLs	−3.40(−3.49, −3.31)	<0.001	−2.49(−2.58, −2.39)	<0.001	−1.47(−1.58, −1.36)	<0.001	−1.44(−1.51, −1.37)	<0.001
YLDs	0.48 (0.44, 0.51)	<0.001	0.62 (0.55, 0.70)	<0.001	0.64 (0.54, 0.75)	<0.001	0.61 (0.51, 0.71)	<0.001
DALYs	−3.33(−3.42, −3.24)	<0.001	−1.87(−1.93, −1.80)	<0.001	−0.90(−0.98, −0.83)	<0.001	−1.09(−1.14, −1.04)	<0.001

DALYs, disability-adjusted life years; YLDs, years lived with disability; YLLs, years of life lost; UI, uncertainty interval; CI, confidence interval.

**Figure 2:**
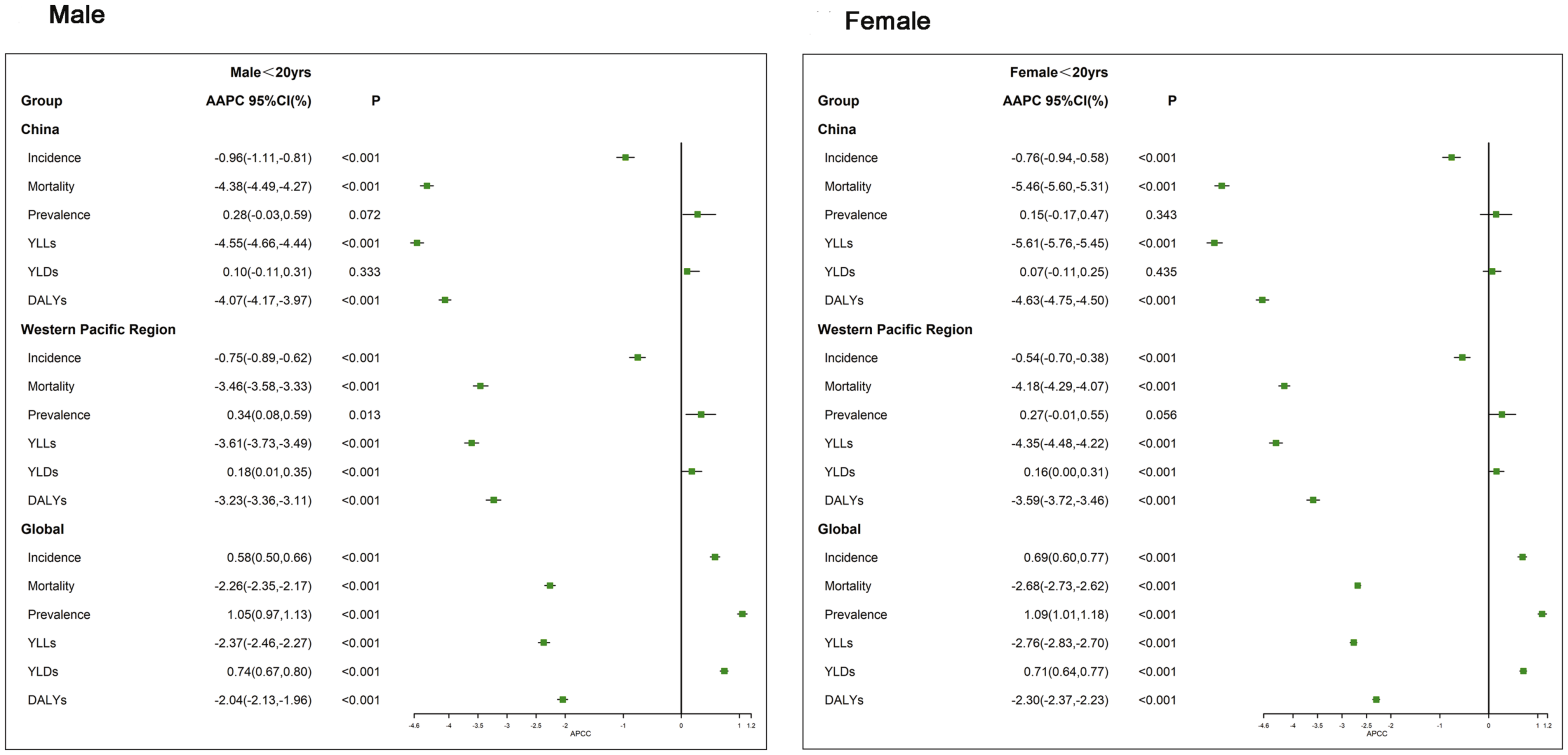
Average annual percentage change (AAPC) of incidence, prevalence, deaths, YLLs, YLDs, and DALYs of CVD in people younger than 20  years stratified by gender for 1990–2019 in China, the Western Pacific Region, and the world.

The AAPC of incidence, prevalence, deaths, YLLs, YLDs, and DALYs due to CVD among people under 20 years of age, stratified by gender from 1990 to 2019 in China, Western Pacific Region, and the world is shown in Figure 2. We found that a reduction in the AAPC of mortality, YLLs, and DALYs for both sexes combined was observed, with a greater reduction among female patients than male patients significantly (all *p* < 0.001). However, at the global level, there was a slower increase in the AAPC of incidence (male patients: AAPC = 0.58%; female patients: AAPC = 0.69%).

**Figure 3:**
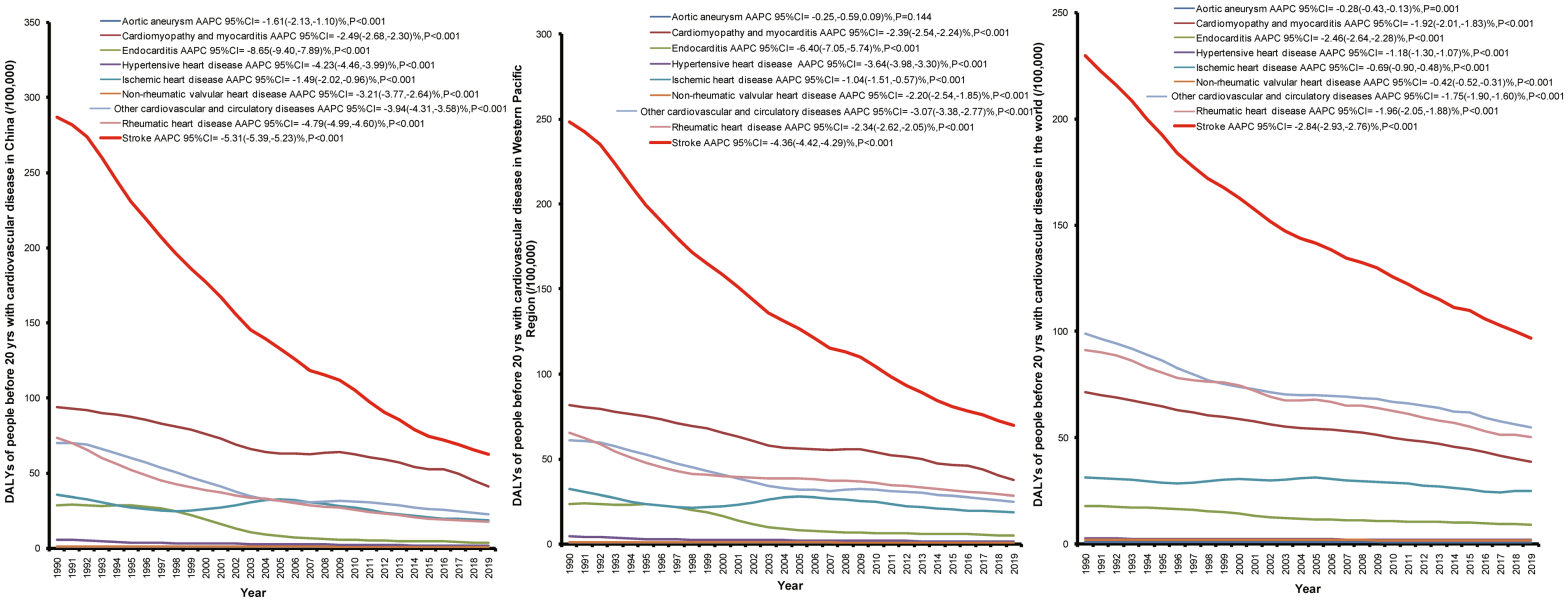
Long-term trends and AAPC values of DALYs among people before 20  years for all subtypes of CVD from 1990 to 2019 in China, the Western Pacific Region, and the world.

Figure 3 displays the long-term trend of DALYs among people under 20 years for all subtypes of CVD from 1990 to 2019. For all subtypes of CVD, the AAPC values showed a downward trend, regardless of the world, the Western Pacific Region, and China. The largest reduction was observed for stroke, such as DALYs in China, which went from 286.89/100,000 in 1990 to 62.51/100,000 in 2019, whereas other subtypes of CVD showed only a slight decrease. The AAPC of DALYs for all subtypes of CVD was significantly decreasing (all *p* < 0.001). In China and the Western Pacific Region, the AAPC in DALYs for endocarditis between 1990 and 2019 was the largest, which was −8.65% (95% CI: −9.40% to −7.89%) and −6.40% (95% CI: −7.05% to −5.74%), respectively.

**Figure 4:**
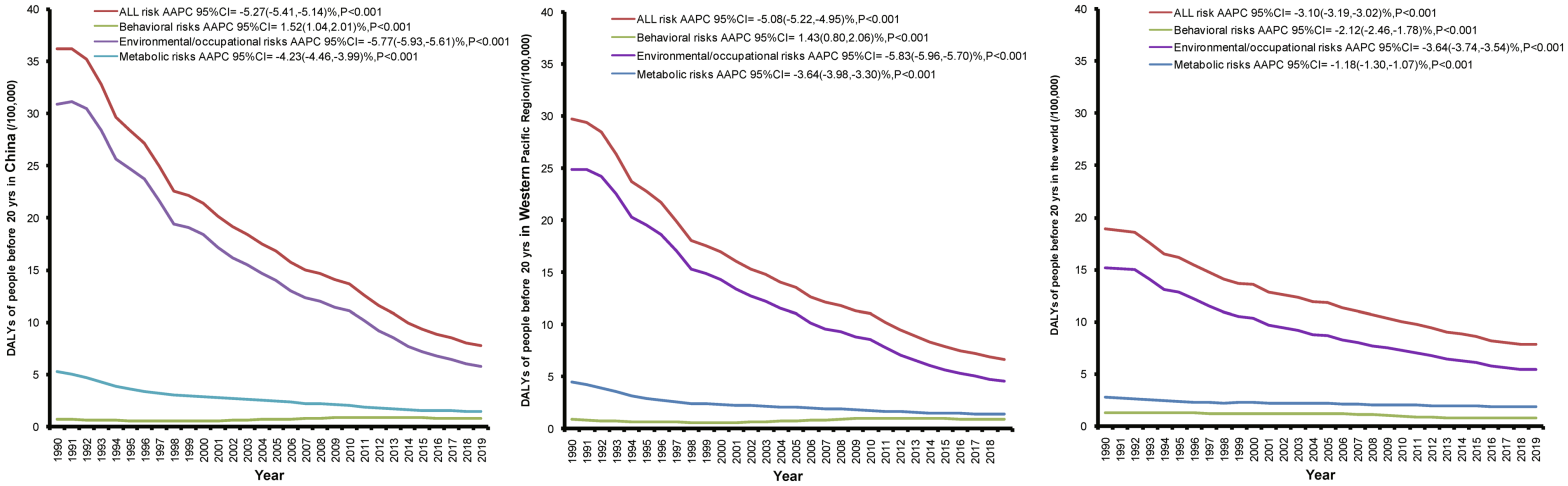
Long-term trends and AAPC values of CVD-related DALYs attributable to risk factors among people under 20 years from 1990 to 2019 in China, the Western Pacific Region, and the world.

Figure 4 illustrates the long-term trend of CVD-related DALYs attributable to risk factors from 1990 to 2019 among people under 20 years of age. We focused on three major risk factors for CVD: behavioral risks, environmental/occupational risks, and metabolic risks. A decline in the DALY rate for all CVD risk factors was observed in China, the Western Pacific Region, and the world from 1990 to 2019, with a significant decrease in environmental/occupational risk factors. China had the largest AAPC values in all CVD risk factors (AAPC = −5.27, 95% CI: −5.41% to −5.14%).

## Discussion

CVD remains the leading cause of premature mortality and rising healthcare costs and is a significant global health problem. Epidemiological studies in terms of the incidence, prevalence, deaths, YLLs, YLDs, and DALYs in CVD burden are sparse because of the overall low incidence of overt CVD among children and adolescents. To our knowledge, this is the first comprehensive and comparative report on the disease burden of CVD and its trends among people under 20 years of age in China, the Western Pacific Region, and the world over a 30-year period from 1990 to 2019. Our results provided several key messages. Globally in 2019, there were 2.37 million incidences of CVD, 16.85 million prevalence of CVD, and 74386.73 deaths due to CVD among people under 20 years of age. The burden and trends of CVD in China, the Western Pacific Region, and the global scale generally showed a downward trend among people younger than 20 years. The decrease in CVD burden is consistent with previous findings and may be attributed to the enforcement of life-saving health policies and interventions, rapid development of medical standards and essential treatment, as well as maternal education (13–15). Pathological evidence of atherosclerosis in young individuals was first identified in young male casualties from the Korean and Vietnam Wars and then further characterized by the Bogalusa Heart Study (16). CVD could begin in childhood and may progressively worsen without any appropriate intervention. Therefore, it is critical to pay attention to the burden of CVD and trends in childhood and actively implement effective interventions.

Findings from this study found that at the global level, the AAPC in the incidence and prevalence of CVD was observed to increase from 1990 to 2019. Chen Huang et al. (17) also revealed that the prevalence of CVD has been increasing in children, adolescents, and young adults in recent decades in developed countries. However, opposite results were observed for the incidence of APCC in China and the Western Pacific region. This discrepancy may be explained by the fact that many countries are included in the analysis of the global data, with different levels of development, medical conditions, distribution of health resources, and perception of CVD. We further observed that with the increase in age, the AAPC values of mortality, YLLs, and DALYs showed a notable downward trend. We speculated that this change was largely related to the global decline in fertility or increased awareness of healthy living in childhood. Comparatively, although there was a decline in the CVD burden, the increase in the absolute incidence of cases of CVD could not be ignored. Furthermore, our results found that the CVD burden showed heterogeneity by gender in China, the Western Pacific Region, and the world. A decrease in the AAPC of mortality, YLLs, and DALYs was significantly higher in female patients than in male patients. This difference may be related to physiological differences, estrogen levels, and lifestyle between male patients and female patients (18). This gender difference should be taken into account by policymakers when planning future strategies and implementing interventions. There were marked differences in the trends of DALYs in terms of its main subcategories, including ischemic heart disease, stroke, endocarditis, and hypertensive heart disease. The AAPC values showed a downward trend, with the greatest decline in stroke. As for CVD subcategories, in clinical settings, stroke has been increasingly recognized as the main single type of atherosclerotic CVD in China, the Western Pacific Region, and the world (5). Although previous studies had reported a substantial increase in the burden of stroke (19), an obvious decline in the AAPC value and trend of stroke were observed in this study. Our results are consistent with a temporal trend in ischemic stroke incidence in younger adults in the Framingham study (20). A population study also observed that stroke due to small vessel occlusion and cardiac embolism may be on the decline (21). Long-term trends in stroke are controversial and may result from differences in the availability and affordability of medications, patient adherence to treatment, quality of healthcare, and appropriate management of post-discharge secondary prevention provided by professionals.

Behavioral, environmental/occupational, and metabolic risk factors are major drivers of CVD. Traditional risk factors such as high blood pressure (22), elevated lipid levels (23), smoking (24), a sedentary lifestyle (25), and being overweight (26) are well known; however, it is important to recognize that these risk factors often develop and begin to detrimentally affect health during childhood (27). In our study, a decline in the DALY rate for all CVD risk factors was observed over the past 30 years among people younger than 20 years, with a significant decrease in environmental/occupational risk factors. During this period of rising metabolic risk exposure, the burden of CVD has been declining, a seemingly paradoxical phenomenon that can be largely explained by the impact of access to care, social determinants of health, cohort effects, and other behavioral, occupational, and environmental risks not quantified here (7, 28, 29). There is growing evidence that environmental influences on an individual’s cardiovascular health begin in childhood and even in the womb (30). As is well known, the early years of life, when behaviors are still being learned, are a great opportunity to educate children about cardiovascular health. It is much easier to teach children healthy habits than to change well-established unhealthy behaviors in adults.

This study is based on the largest epidemiological dataset to date and is the first to provide systematic estimates of the CVD burden and trend of people under 20 years, stratified by gender and subtypes, as well as CVD-related DALYs attributable to risk factors in China, the Western Pacific Region, and the world from 1990 to 2019. However, it has some limitations. First, as part of the GBD study, all limitations of the GBD methodology affected this study, which have been described previously (31–33). Second, within the age range of the analysis, we did not make age-standardized adjustments for the corresponding indicators. Third, we have only roughly estimated three main risk factors for CVD-related DALYs. In addition, a comprehensive assessment of the burden of disease should also include economic, family, and social aspects, so multidimensional analysis can be considered to improve the accuracy of the results.

## Conclusion

This study has evaluated the burden of CVD and its trends among people under 20 years, stratified by gender and subtypes in China, the Western Pacific Region, and the world from 1990 to 2019. Overall, the CVD burden saw a substantial decline among children and adolescents, although variability across countries was present. Targeted considerations were needed to integrate primary prevention and take effective measures in childhood to reduce the future burden of CVD in adults.

## Data availability statement

The datasets presented in this study can be found in online repositories. The names of the repository/repositories and accession number (s) can be found at: https://vizhub.healthdata.org/gbd-results/.

## Author contributions

YZ, XL, and ZL conceived and designed the experiment. YZ, CL, ML, and XL analyzed the data. ML, XX, JW, and ZL provided significant advice and consultation. YZ wrote the manuscript. All authors contributed to the article and approved the submitted version.

## Funding

This study was supported by the Basic Research Program of Shanxi Province (Free exploration) project (20210302123216 to YZ), the Science and Technology Innovation Project of Higher Education Institutions in Shanxi Province (2021L221 to YZ), the Special Disease Construction Project of Pudong Health and Family Planning Commission of Shanghai (Grant No. PWZzb2022-20 to ZL), Discipline Construction Project of Pudong Health and Family Planning Commission of Shanghai (Grant No. PWYts2021-02 to ZL); the General Program of Health Bureau of the Shanghai Pudong New Area (PW2021A-68 to ZL), the Shanghai Health Medical College Faculty Teaching Project (ZPJXKT-21-11 to ZL), the General Program of Health Bureau of the Shanghai (202150015 to ZL), and the Shanghai Science and Technology Development Foundation (53310000501772180D to CL). The funder had no role in study design, data collection, data analysis, data interpretation, or writing of the report. All authors had full access to all data in the study and had responsibility for the decision to submit for publication.

## Conflict of interest

The authors declare that they have no known competing financial interests or personal relationships that could have appeared to influence the article reported in this study.

## Publisher’s note

All claims expressed in this article are solely those of the authors and do not necessarily represent those of their affiliated organizations, or those of the publisher, the editors and the reviewers. Any product that may be evaluated in this article, or claim that may be made by its manufacturer, is not guaranteed or endorsed by the publisher.
